# Cyto-histologic discordancy in patients undergoing thyroidectomy at Aga Khan University Hospital

**DOI:** 10.11604/pamj.2019.32.135.16524

**Published:** 2019-03-21

**Authors:** Moses Barasa, Abdulkarim Abdallah

**Affiliations:** 1 General Surgery, Aga Khan University, Nairobi, Kenya; 2 Consultancy Surgery, Aga Khan University, Nairobi, Kenya

**Keywords:** Cytology, histology, discrepancy

## Abstract

Fine needle aspiration cytology (FNAC) findings are the basis upon which decision and type surgery is made. Therefore the diagnostic accuracy and utility of FNAC being such an integral tool in management of thyroid lesions must be evaluated for cyto-histologic discrepancy from time to time as a quality assurance measure. The objective was to compare thyroid fine-needle aspiration cytology (FNAC) with final histopathological findings at in patients undergoing thyroidectomy. This was a cross-sectional retrospective study at Aga Khan university hospital, Nairobi. Cyto-histologic discrepancy was found in 19(25%) of cases with false positive and negative rates at 9% and 16%. False positive results constituted 7(9%) while 12(16%) were false negative findings. The higher cyto-histological discordancy than seen previous studies could be due to sampling error and cytological mis-interpretation. Our study found higher than expected cyto-histologic discrepancy.

## Introduction

The prevalence of thyroid nodules ranges from 4% to 10% in the general adult population. The majority of clinically diagnosed thyroid nodules are non-neoplastic; only 5%-30% are malignant and require surgical intervention [1]. Total thyroidectomy is recommended for malignant thyroid disease while thyroid conservation surgery is done for benign symptomatic euthyroid disease. Moreover, patients with asymptomatic euthyroid disease undergo watchful waiting [1, 2]. Total Thyroidectomy carries risks including injury to recurrent laryngeal nerve, hypoparathyroidism, hypothyroidism and need for life time hormone replacement therapy and therefore it should only be done for patients who need it. These risks are even higher for completion thyroidectomy due to post-surgical adhesions and distorted surgical anatomy [2-4]. Decision to operate and type of surgery depend on the pre-operative cytological diagnosis obtained via FNAC [3, 4]. FNAC is a minimally invasive, accurate and cost-effective diagnostic tool for differentiating benign from malignant thyroid lesions thus providing basis for decisions on management options. Whereas FNAC has a high degree of proven sensitivity (90%) and specificity (96%), it's however associated with a false positive and negative interpretation that averages from 1 to 6% respectively [4, 5]. Papanicolou international cytopathology society guidelines state that acceptable false negative and positive rates should be less than 2% and 3%, respectively [6]. Continuous histo-cytological correlation is an important quality control assurance measure that allows laboratories to improve their quality to conform to internationally accepted standards. The aim of this study was to compare thyroid fine-needle aspiration cytology (FNAC) with final histopathological findings at Aga Khan University Hospital as quality control assurance measure.

## Methods

This was retrospective cross-sectional study of patients with goitre who had pre-operative FNAC with subsequent histological evaluation post thyroidectomy at Aga Khan University Hospital between January to December 2017. Patient files were retrieved and information about age, sex, clinical diagnosis, FNAC diagnosis, surgical procedure and definitive tissue histology were recorded. Excluded from the study were all patients whose FNAC and/or histology results were missing, incomplete or those whose FNAC were not done at Aga khan pathology laboratory. Confidentiality of all patient records was maintained and Permission from Aga Khan University research committee approved use of hospital medical records data and publishing of this audit results.

## Results

A total of 103 files were sampled of which 76 met the inclusion criteria. Of the 76 cases subjected to cyto-histologic examination 57(75%) were concordant while 19(25%) were discordant. Further, of the cyto-histologic discrepant cases, false positive results constituted 7(9%) while 12(16%) were false negative findings. The correlation of cytological and histopathological diagnoses in this audit is summarized in Table 1.

**Table 1:** Correlation of cytological and histopathological diagnosis; FNAC diagnoses on Y axis correlated with corresponding histopathological diagnoses on X-axis

Histology FNAC	Colloid goitre	Hashomoto’s thyroiditis	Adenomatous goitre	Follicular adenoma	Hurthle cell neoplas	Follicular carcinoma	Papillary carcinoma	Anaplastic carcinoma	Total
Colloid nodule/goitre	47	1	1				7		56
Hashimoto’s thyroiditis		1					1		2
Follicular neoplasm	2			1	1	4	2		10
Hurthle cell neoplasm				1					1
Papillary carcinoma	3						3		6
Medullary carcinoma									0
Anaplastic malignancy	1								1
Total	54	2	1	2	1	4	13		76

## Discussion

Cyto-histologic discrepancy was found in 19(25%) of cases with false positive and negative rates at 9% and 16%. This is higher than previous studies in which false positive and negative rate of up to 7% and 11% respectively [7, 8]. Previous studies have cited that this could be due to sampling error and cytological mis-interpretation [8, 9]. All types of false negative and false positive results cause concern because the reliability of cytology is in question. According to Bakhos, S M *et al* and Sharma C *et al*, false negative cases are encountered when there are no recognizable diagnostic cells in the smear because of sampling or processing error therefore overlooking of malignancy in favour of benign lesions. Conversely, false positive diagnosis is more the result of misinterpretation of the nature of benign cell than a sampling error. They are usually encountered in Hashimoto's thyroiditis, follicular/parathyroid/atypical adenoma and colloid nodules [10, 11]

## Conclusion

The rate of cyto-histological discrepancy in our study is higher than the recommended values set by the Papanicolaou Task Force on Standards of Practice thus raising an important quality assurance issue on diagnostic utility of FNAC in thyroid lesions at Aga Khan University Hospital. Subsequently, we have designed a follow-up study to determine factors associated with this high rate of cyto-histologic discordancy with aim of formulating possible practical solutions.

### What is known about this topic

- Fine needle aspirate cytology of the thyroid is the gold standard, most sensitive, specific, non-invasive, cost-effective and efficient method of differentiating benign and malignant thyroid nodules.

### What this study adds

- Diagnostic accuracy of FNAC in thyroid lesions varies from institution to institution;
- It's important for individual institutions to ascertain and compare their discrepant cytologic/histopathologic rates to the set gold standard by Papanicolaou Task Force on standards of practice;
- Identification of possible causes and solutions to this cytohistological discrepancy will improve diagnostic accuracy of fnac in thyroid nodules.

## Competing interests

The authors declare no competing interests.
